# Abdominal Pain in the Elderly: An Unusual Case of Chronic Mesenteric Ischemia in the Emergency Department

**DOI:** 10.5811/cpcem.2019.5.41886

**Published:** 2019-07-01

**Authors:** Julieta I. Lacey, Robert M. Hughes, Vicki Noble

**Affiliations:** University Hospitals Cleveland Medical Center, Department of Emergency Medicine, Cleveland, Ohio

## Abstract

Chronic mesenteric ischemia (CMI) is a rare cause of abdominal pain with the potential for significant morbidity and mortality. An infrequently described complication of CMI is acalculous cholecystitis. Historically, acalculous cholecystitis is thought to be multifactorial and usually occurs in the setting of severe illness. In CMI, the etiology is more likely chronic ischemia to the gallbladder leading to inflammation. We present a case of acalculous cholecystitis that presented insidiously in a patient with CMI.

## INTRODUCTION

Chronic mesenteric ischemia (CMI) is a rare, but potentially fatal, cause of abdominal pain. CMI refers to splanchnic hypoperfusion involving at least two of the following: the celiac artery, superior mesenteric artery, and inferior mesenteric artery.^2^ In a recent study, the prevalence of symptomatic CMI was reported to be as low as 0.03% despite the growing prevalence of mesenteric artery stenosis.^3^ CMI is typically secondary to atherosclerosis and most commonly involves the celiac artery.^1^,^3^ A usual presentation consists of intestinal angina and progressive weight loss.

One study shows that significant stenosis, especially greater than 70%, within the splanchnic vessels can lead to the development of collateral vessels in order to prevent ischemia to the bowels.^4^ This angiogenesis, along with the body’s intrinsic collateral vessels, serves to protect the remainder of the splanchnic organs from significant ischemia as well. One notable exception is the gallbladder, which receives its blood supply primarily from the cystic artery, a terminal branch of the celiac artery.^6^ To our knowledge, there is no evidence to support the formation of collaterals to the gallbladder in CMI.

Acalculous cholecystitis, an infrequently described complication of CMI, may present with the typical symptoms of CMI and should be considered, especially in the setting of right upper quadrant pain, leukocytosis and elevated inflammatory markers in patients with risk factors for vascular disease. While acute acalculous cholecystitis is most often described in the critically ill, we describe a case of acute acalculous cholecystitis that presented with marked leukocytosis and hypotension in an otherwise well appearing, elderly female with underlying CMI.

## CASE REPORT

A 76-year-old Caucasian female with a past medical history significant for hyperlipidemia and poorly controlled hypertension was sent to the emergency department (ED) by her outpatient cardiologist due to abnormal labs. Her labs were most significant for a severe leukocytosis and hypokalemia. On her arrival to the ED, she was grossly asymptomatic but reported a two-week history of generalized fatigue and intermittent lightheadedness. On review of systems, she also endorsed a 10-pound weight loss over the prior six months and episodic generalized abdominal pain associated with nausea and vomiting over the prior year.

On exam, she was hypotensive to 83/42 millimeters of mercury with otherwise normal vital signs. She was awake, alert, in no acute distress, and in general was very comfortable and well appearing. She did not demonstrate any evidence of hypoperfusion on exam such as altered mental status, dizziness, or cold extremities. Her abdomen was soft and non-distended with only mild tenderness to palpation in the suprapubic region but no guarding or rebound. Her exam was otherwise notable for a diminished left radial pulse and bilaterally diminished dorsalis pedis pulses.

Routine investigations revealed a white cell count of 49.1/liters (L) (4.4–11.3×10^9^/L) with a left shift and neutrophil predominance, potassium of 2.5 millimoles (mmol)/L (3.5–5.3mmol/L), sodium of 127 mmol/L (136–145mmol/L), blood urea nitrogen of 31 milligrams per deciliter (mg/dL) (6–23 mg/dL), creatinine of 1.53 mg/dL (0.50–1.05 mg/dL), C reactive protein of 31.44 mg/dL (<1.00) and lactate of 3.7 mmol/L (0.4–2.0 mmol/L). Her liver function tests were within normal limits. Urinalysis and chest radiograph were obtained for an infectious workup and were both unremarkable.

After a period of observation, an abdominal computed tomography (CT) was obtained given her history of chronic abdominal pain and persistent concern for infectious process with no clear source. The CT showed gallbladder wall thickening and edema with pericholecystic edema and a contained perforation of the gallbladder wall near the fundus (Image).

Interval CT angiogram showed severe atherosclerotic changes of the abdominal aorta and its branches with complete occlusion of the celiac artery origin and diminutive flow in its distal branches from collateral vessels, severe stenosis of the superior mesenteric artery, and mild stenosis of the inferior mesenteric artery. There was mild wall thickening of the ascending colon and transverse colon.

## DISCUSSION

Acalculous cholecystitis is an infrequent, acute inflammatory disease of the gallbladder, thought to be multifactorial in etiology, which most often occurs in critically ill patients.^6^ Acalculous cholecystitis as a result of CMI is an especially rare complication with only a handful of reported cases in the literature. Melo et al. reported a case of acute acalculous cholecystitis in a patient with severe atherosclerosis and CMI. This patient’s presentation consisted of acute onset, right upper quadrant pain while admitted to the hospital for a planned revascularization. He underwent a simultaneous cholecystectomy and elective revascularization of his aortic and visceral occlusive disease. Other case reports present instances of acute acalculous cholecystitis in patients with critical illness causing systemic inflammation such as a severe infection, systemic lupus erythematosus, or burns.^7^,^8^,^9^ Our case contrasts with the above case reports in that our patient’s abdominal pain was chronic and not present on the day of her presentation. A high index of suspicion is required to consider acalculous cholecystitis as a cause of her severe leukocytosis in this otherwise well-appearing female with a benign abdominal exam.

**CPC-EM Capsule**

What do we already know about this clinical entity?

A rare complication of chronic mesenteric ischemia (CMI), acalculous cholecystitis, is most often seen in critically ill patients.

What makes this presentation of disease reportable?

Acalculous cholecystitis in the setting of mesenteric ischemia is a rare cause of chronic abdominal pain, a common chief complaint in the emergency department.

What is the major learning point?

Acalculous cholecystitis is a rare complication of CMI that should be considered in elderly patients with abdominal pain and risk factors for vascular disease.

How might this improve emergency medicine practice?

Early recognition and diagnosis of acalculous cholecystitis in patients with post-prandial abdominal pain may expedite treatment and improve outcomes.

Although it is understood that the pathogenesis of acalculous cholecystitis is multifactorial, ischemia has been more recently recognized as an important risk factor for its development.^6^ Our case demonstrates the development of acalculous cholecystitis in a patient with no identified risk factors other than peripheral vascular disease as demonstrated by unequal and diminished peripheral pulses on exam and by areas of severe stenoses on her subsequent CT angiogram. Her history of poorly controlled hypertension was also suggestive of renal artery stenosis. She had not had any other acute illness in the weeks prior to predispose her to systemic inflammation and to the acute irritation and subsequent perforation of her gallbladder in the absence of cholelithiasis.

It is clear that acalculous cholecystitis can develop in an array of clinical settings. Systemic illness has been established, and frequently cited, as a known risk factor. However, given the gallbladder’s terminal blood flow and susceptibility to ischemia, acalculous cholecystitis should be recognized as a possible complication of mesenteric atherosclerosis and specifically CMI.

## CONCLUSION

The patient was treated prophylactically with piperacillin/tazobactam and admitted to the surgical service for a laparoscopic cholecystectomy. She underwent the procedure without complications and was discharged home. She then followed up with her cardiologist to discuss the findings on her CT angiogram and her CMI. She was feeling well after her cholecystectomy, and because of her lack of symptoms they decided to pursue conservative management of her CMI rather than revascularization.

## Figures and Tables

**Image:**
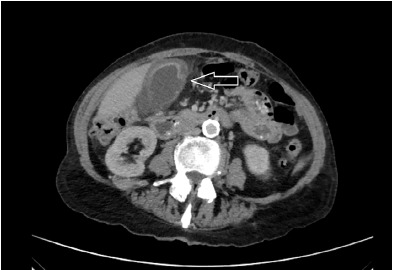
Abdominal computed tomography scan with contrast showing cholecystitis with evidence of gallbladder wall perforation (white arrow). Gallbladder wall appears thickened and edematous and there is a moderate amount of pericholecystic fluid and inflammatory stranding. In addition, there is an apparent discontinuity in the gallbladder wall.
